# Green Synthesis of Silver Nanoparticles Using *Astragalus tribuloides* Delile. Root Extract: Characterization, Antioxidant, Antibacterial, and Anti-Inflammatory Activities

**DOI:** 10.3390/nano10122383

**Published:** 2020-11-29

**Authors:** Majid Sharifi-Rad, Pawel Pohl, Francesco Epifano, José M. Álvarez-Suarez

**Affiliations:** 1 Department of Range and Watershed Management, Faculty of Water and Soil, University of Zabol, Zabol 98613-35856, Iran; 2 Department of Analytical Chemistry and Chemical Metallurgy, Faculty of Chemistry, Wroclaw University of Science and Technology, Wyspianskiego 27, 50-370 Wroclaw, Poland; 3 Dipartimento di Farmacia, Università “G. d’Annunzio” Chieti-Pescara, Via dei Vestini 31, 66100 Chieti Scalo, Italy; fepifano@unich.it; 4 King Fahd Medical Research Center, King Abdulaziz University, Jeddah 21589, Saudi Arabia; 5 AgroScience & Food Research Group, Universidad de Las Américas, Quito 170125, Ecuador

**Keywords:** silver nanoparticle, biomedical application, green synthesis, *A. tribuloides*

## Abstract

Today, the green synthesis of metal nanoparticles is a promising strategy in material science and nanotechnology. In this research, silver nanoparticles (AgNPs) were synthesized through the high-efficient, cost-effective green and facile process, using the *Astragalus tribuloides* Delile. root extract as a bioreduction and capping agent at room temperature. UV–Vis spectroscopy was applied for the investigation of the reaction proceedings. To characterize the greenly synthesized AgNPs, Fourier-transform infrared spectroscopy (FTIR), X-ray diffraction spectroscopy (XRD), and transmission electron microscopy (TEM) analyses were utilized. In addition, the total phenolics and flavonoids contents, antioxidant, antibacterial, and anti-inflammatory activities of the greenly synthesized AgNPs and the *A. tribuloides* root extract were evaluated. The results indicated that the AgNPs had spherical morphology and crystalline structure with the average size of 34.2 ± 8.0 nm. The total phenolics and flavonoids contents of the greenly synthesized AgNPs were lower than those for the *A. tribuloides* root extract. The resultant AgNPs exhibited the appropriate antioxidant activity (64%) as compared to that for the *A. tribuloides* root extract (47%). The antibacterial test approved the higher bactericidal activity of the resulting AgNPs on the Gram-positive and Gram-negative bacteria in comparison to the *A. tribuloides* root extract. Considering the anti-inflammatory activity, the greenly synthesized AgNPs showed a stranger effect than the *A. tribuloides* root extract (82% versus 69% at 500 μg/mL). Generally, the AgNPs that were fabricated by using the *A. tribuloides* root extract had appropriate antioxidant, antibacterial, and anti-inflammatory activities and, therefore, can be considered as a promising candidate for various biomedical applications.

## 1. Introduction

Nanotechnology is considered one of the most important fields of study in material science [1]. Due to large surface-area-to-volume ratios, nanoparticles (NPs) have amazing physical and chemical characteristics as compared to their bulk materials [2,3,4]. Nanoparticles can be produced through chemical, physical, or biological procedures. The biological procedure is more promising as compared to physical and chemical methods because it is economical, flexible, and eco-friendly [5]. In this method, microorganisms or medicinal plants are used for the nanoparticles’ production [6]. However, the previous reports have shown the synthesis of nanoparticles carried out with the microorganisms is slower in comparison to the plant-based synthesis [7]. Moreover, many researchers prefer the synthesis of plant-mediated nanoparticles to the microbial-based synthesis because it is economical and does not require a long-term storage of the microbial cultures [8]. The study, design, synthesis, and characterization of various metallic nanoparticles for the treatment and targeting of many diseases have received much attention recently [9,10]. From the noble metallic nanomaterials, the silver nanoparticles (AgNPs) differ due to their attractive physical and chemical characteristics related to different biological systems [11]. The AgNPs are under a wide study, due to their participation in various fields, including food packaging, medicine, pharmacology, and healthcare [12,13]. The antioxidant, antimicrobial, and anti-inflammatory effects of the AgNPs are well-documented in scientific reports [14,15]. Reduced Ag is toxic to microorganisms because it has the ability to damage their cell walls and disrupt their appropriate functions and development. This is because the released Ag ions interact with macromolecules like deoxyribonucleic acid (DNA) and proteins in these cells. For example, they prevent the synthesis of protein, reduce the permeability of the membranes, and ultimately lead to the cell death [3,16]. Because of their small size and the large surface-area-to-volume ratio, the AgNPs might show the higher biological activity than that exerted by ionic Ag. 

The genus *Astragalus* belongs to the Fabaceae family and is mainly distributed in Europe, North Africa, Asia, and the Mediterranean. Since ancient times, *Astragalus* roots have been applied as a main drug in folk medicine in Russia, Bulgaria, and China, to enhance the immune system and decrease inflammation [17,18]. The roots are applied for treating the eyes, tumors, throat, liver, and chest and back pains. They are also used to regenerate the tissues and heal wounds [19]. Phytochemical investigations on the *Astragalus* species indicated that their roots are rich in saponins, phenolics, flavonoids, and polysaccharides [20,21]. The antibacterial, antioxidant, and anti-inflammatory effects of the *Astragalus* plants have been reported in many studies [22,23].

Given the importance of the green synthesis of the AgNPs that have the specific biological activity and could potentially be used for the medicinal purposes, the current study represents a fast, very easy one-step green synthesis of the AgNPs by the *A. tribuloides* Delile. root extract. Antioxidant, antibacterial, and anti-inflammatory effects of the resultant nanomaterial were investigated. To the extent of our knowledge, this is the first study on the green synthesis of the AgNPs by the root extract of the studied medicinal plant.

## 2. Materials and Methods

### 2.1. Plant Material

The *A. tribuloides* roots were collected in June 2020, from Iranshahr rangelands, Sistan and Baluchistan Province, Iran. The voucher specimen (No. 9938) was deposited in the herbarium of the Department of Rangeland and Watershed Management, University of Zabol, Zabol, Iran. Fresh roots were washed, air-dried, and converted into a powder, using an electric grinder (Pars khazar, Iran). For the preparation of the plant extract, 5 g of the root powder was treated with 100 mL of ethanol (98%), for 24 h, at room temperature, and then filtered by a Whatman filter paper No.1. The resulting extract was stored at 4 ± 1 °C, as the stock solution, and used directly for the green synthesis of the AgNPs. It was also evaporated to dryness, under vacuum, and stored at 4 ± 1 °C until the further analysis.

### 2.2. Green Synthesis of the AgNPs 

For the green synthesis of the AgNPs, 10 mL of the *A. tribuloides* root extract was added into 90 mL of a 1 mmol L^−1^ aqueous solution of silver nitrate (AgNO_3_) (Merck, Darmstadt, Germany). The resultant mixtures were kept at room temperature, for different times (up to 240 min), and then centrifuged at 15,000× *g*, for 20 min, for separation of the green-synthesized AgNPs. To remove other impurities that settled down with the synthesized NPs, the resulting pellets were washed three times, using deionized water, and allowed to dry at room temperature.

### 2.3. Characterization of the Green-Synthesized AgNPs

The formation of the AgNPs during their green synthesis was monitored by UV–Vis absorption spectroscopy. The absorbance spectra of the reaction mixtures were acquired by a Shimadzu UV–Vis spectrophotometer, model UV-1800 (Shimadzu Corporation, Kyoto, Japan), in the range of 200–800 nm, at different time intervals. For the blank sample, the distilled water was considered.

FTIR spectroscopy was performed, to recognize the functional groups of the plant extract compounds, which were capped at the surface of the AgNPs and could participate in their synthesis. For the preparation of the samples, the dried *A. tribuloides* root extract and the AgNPs were used. To characterize the samples, a Nicolet 800 FTIR spectrometer (Nicolet, Madison, WI, USA), by the absorbance mode and using a potassium bromide (KBr) pellet, was used. For this aim, samples were blended with the KBr powder and converted into the pellet by pressing it; the pellet was subjected to FTIR spectroscopic analysis in the spectral range of 4000 to 500 cm^−1^, with the 4 cm^−1^ resolution, as explained by Sharifi-Rad and Pohl [3].

The crystal structure of the greenly synthesized AgNPs was investigated by using X-ray diffraction (XRD) by a Cu-Kα radiation (λ = 1.54 Å), in the 2θ ranging from 20° to 90°, at a 0.026°/min scanning rate. A Siemens X-ray diffractometer, model D5000 (Munich, Germany), was used for that aim. The average crystalline size of the greenly synthesized AgNPs was determined by using the Debye–Scherrer’s equation, as explained by Bagherzade et al. [24].

The size distribution and the morphological characteristics of the greenly synthesized AgNPs were evaluated by a Philips GM-30 transmission electron microscope (Hillsboro, OR, USA), at an accelerating voltage of 120 kV, with a resolution of 2.5 Å, as suggested by the TEM instrument supplier. For the preparation of the samples, the cleaned AgNPs were re-dispersed in a solution, and one drop of this solution was placed on a copper grid, followed by the evaporation of solvent under an infrared lamp. The Digimizer software (version 4.1.1.0) was applied for the measurement of the particles size distribution, using the TEM images.

### 2.4. In Vitro Biological Activities

#### 2.4.1. Total Phenolics Content Determination

The total phenolics content (TPC) in the *A. tribuloides* root extract and greenly synthesized AgNPs samples was measured by the Folin–Ciocalteau reagent, as explained by Sharifi-Rad et al. [25], with slight modifications. For this purpose, 1 mL of the freshly prepared Folin–Ciocalteu reagent (0.2 mol L^−1^) was separately mixed with 0.5 mL of the *A. tribuloides* root extract and greenly synthesized AgNPs at various concentrations (100, 200, 300, 400, and 500 μg mL^−1^). Then 0.8 mL of sodium carbonate (7.5% of Na_2_CO_3_) was added to the mixture after 5 min. The resultant mixtures were maintained in the dark condition at room temperature and the absorbance of the reaction mixtures were recorded at 760 nm against the reagent blank after 2 h with a Shimadzu UV–Vis spectrophotometer, model UV-1800. Gallic acid was applied for the preparation of the standard solutions of the calibration curve at the same operating conditions. The TPC of the *A. tribuloides* root extract and the greenly synthesized AgNPs was measured by the standard curve, and the results are presented as μg gallic acid equivalents (GAE).

#### 2.4.2. Total Flavonoid Content Determination

The total flavonoid content (TFC) of the *A. tribuloides* root extract and greenly synthesized AgNPs was measured based on the colorimetric assay explained by Sharifi-Rad et al. [25], but with slight modifications. Briefly, 0.5 mL of the *A. tribuloides* root extract and the AgNPs at various concentrations (100, 200, 300, 400, and 500 μg/mL) was separately mixed with 1.5 mL of methanol, 0.1 mL of aluminum chloride (AlCl_3_) solution (10%), 0.1 mL of 1 mol/L potassium acetate, and 2.8 mL of distilled water, and the resultant mixtures were maintained at room temperature. After 30 min, the absorbance of the mixtures was determined by a UV–Vis spectrophotometer, at 415 nm. The TFC was measured by a quercetin standard curve, and the results are presented as μg quercetin equivalents (QE).

#### 2.4.3. DPPH Free-Radical Scavenging Assay

The antioxidant activity of the *A. tribuloides* root extract and the greenly synthesized AgNPs was evaluated as the radical scavenging capacity against 2,2-diphenyl-1-picrylhydrazyl radical (DPPH), as explained by Sharifi-Rad et al. [26]. Briefly, various concentrations of the *A. tribuloides* root extract and the greenly synthesized AgNPs (100, 200, 300, 400, and 500 μg/mL) were mixed separately with a DPPH solution (0.1 mmol L^–1^) and then left in the dark, at room temperature (25 ± 1 °C), for 30 min. The absorbance of the resultant solutions was measured by a Shimadzu UV–Vis spectrophotometer, at 517 nm, against a control (consist of solvent and DPPH). The butylated hydroxyanisole (BHA) was used as a positive control. The scavenging percentage of free-radical DPPH was determined by using the following formula:

DPPH inhibition (%) = [(Absorbance _(Control)_ − Absorbance _(sample)_)] × 100

#### 2.4.4. Antibacterial Assays

The antibacterial activity of the *A. tribuloides* root extract and greenly synthesized AgNPs was investigated on four bacteria strains, namely *Bacillus cereus* (ATCC 11778), *Staphylococcus aureus* (ATCC 25923), *Shigella flexneri* (ATCC 12022), and *Escherichia coli* (ATCC 25922). The bacteria were obtained from Iranian Research Organization for Science and Technology (IROST).

##### Disc Diffusion Method

The antibacterial effect of the *A. tribuloides* root extract and greenly synthesized AgNPs was assayed by using the agar disc diffusion method [27]. Briefly, 100 μL of a bacteria suspension (containing 1.5 × 10^8^ CFU/mL of bacteria) was dispersed on Müller–Hinton agar plates. Then, the sterilized paper discs (6 mm in diameter) were impregnated with 20 µL of the *A. tribuloides* root extract or the greenly synthesized AgNPs suspension (500 µg/mL). The disks were placed on the surface of Müller–Hinton agar plates. Gentamicin (10 µg/disk) was used as a positive control. For diffusing the active compounds in the medium, the plates were kept at 4 °C for 2 h. After that, all the plates were incubated at 37 °C for 24 h. The antibacterial activity was then investigated by measuring the clear zones of inhibition to the nearest millimeter (mm). 

##### Determination of the Minimum Inhibitory Concentration (MIC)

The MIC was determined by using the micro-broth dilution method in polystyrene 96-well plates, as described by the Clinical and Laboratory Standards Institute [28]. The concentrations of the *A. tribuloides* root extract and the greenly synthesized AgNPs used for MICs were ranged from 500 to 3.9 µg/mL. Then, 50 µL of the Müller–Hinton Broth and 50 µL of the various concentrations of the *A. tribuloides* root extract were dumped into each well. To the second polystyrene 96-well plate, 50 µL of the Müller–Hinton Broth and 50 µL of the various concentrations of the greenly synthesized AgNPs were added to the wells. Finally, 50 µL of a suspension containing 1.5 × 10^8^ CFU/mL of bacteria was added to the wells. Plates were incubated at 37 °C for 24 h. The medium containing bacteria and the pure medium were intended as positive and negative controls, respectively. The lowest concentrations of the *A. tribuloides* root extract or the AgNPs that showed no visible growth of the studied bacteria were considered as the representative MIC values.

##### Determination of the Minimum Bactericidal Concentration (MBC)

The MBC was measured according to the protocol of the Clinical and Laboratory Standards Institute [28]. From each well of the micro-broth assay, for which bacterial growth was not visible, 50 μL was subcultured on the Müller–Hinton agar plates and incubated for 24 h at 37 °C. The lowest concentrations of the *A. tribuloides* root extract or the greenly synthesized AgNPs that exhibited no bacterial growth were intended as the MBC.

##### Time-Kill Test of the Greenly Synthesized AgNPs

The time-kill test was based on the MIC values, found previously in the micro-broth dilution assay, and carried out using the procedure explained by Souza et al. [29], with slight modifications. The greenly synthesized AgNPs was diluted with the Müller–Hinton broth medium (containing 1.5 × 10^8^ CFU/mL inoculum), to achieve their final concentrations of 0 × MIC, 0.5 × MIC, 1 × MIC, 2 × MIC, and 4 × MIC for each bacterial strain, and incubated at 37 °C. At various exposure-time intervals (0, 0.5, 1, 2, 4, and 6 h), 0.1 mL of the medium was serially diluted in a phosphate buffered saline (PBS) solution (1%) and plated onto the Müller–Hinton agar. The plates were incubated for 24 h, at 37 °C. The results are presented in the log_10_ CFU/mL scale.

#### 2.4.5. Anti-Inflammatory Activity

##### Inhibition of the Protein Denaturation

The protein denaturation method was used for the anti-inflammatory effect of the *A. tribuloides* root extract and the greenly synthesized AgNPs, as reported by Sahoo et al. [30]. Briefly, 2 mL of the *A. tribuloides* root extract or the greenly synthesized AgNPs (at concentrations of 100, 200, 300, 400, and 500 μg/mL) was added to 2.8 mL of the phosphate buffered saline solution (pH 6.4) and blended with 0.2 mL of an egg albumin (obtained from fresh hen’s egg) solution. The reaction mixtures were incubated in a water bath (37 ± 2 °C), for 20 min, and then heated at 70 °C, for 5 min. After cooling, the turbidity was determined by a UV–Vis spectrophotometer, at 660 nm. A phosphate buffer solution was applied as the control. The inhibition of the protein denaturation (%) was determined by the following formula:

Inhibition of protein denaturation (%) = 100 × [1 − Absorbance _(sample)_/Absorbance _(Control)_]

### 2.5. Statistical Analysis

All the experiments were carried out in triplicate. The statistical software package SPSS v. 11.5 (IBM Corporation, Armonk, NY, USA) was used for the data analysis. To compare any significant differences among groups, the analysis of variance (ANOVA) test and the Duncan’s multiple range test were utilized. All data were expressed as means values ± standard deviations (SD).

## 3. Results and Discussion

### 3.1. Visual Approval of the Green Synthesis of AgNPs

**Figure 1:**
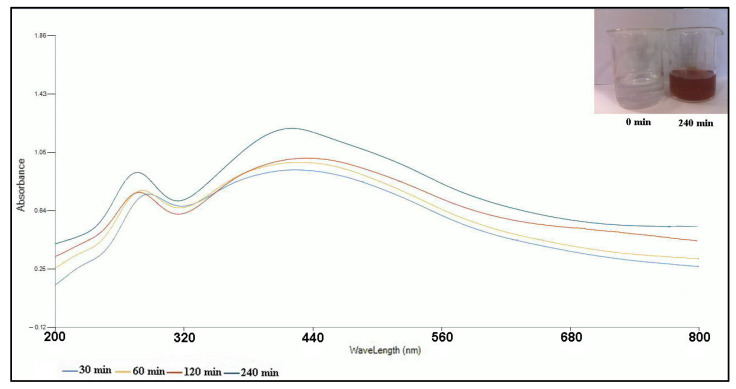
UV–Vis spectra of the greenly synthesized AgNPs by the *A. tribuloides* root extract at various time intervals. The color change of the reaction mixture is also shown.

In the current study, the bioreduction of the Ag (I) ions into the AgNPs was successfully performed by the *A. tribuloides* root extract applied as a bioreducing agent. The AgNPs synthesis was started at room temperature by adding 10 mL of the *A. tribuloides* root extract to 90 mL of an aqueous solution of AgNO_3_ (1 mmol L^–1^). The AgNPs formation was approved by a color change of the colorless AgNO_3_ solution; it changed to dark brown [13] (Figure 1). This indicated that the useful phytochemical constituents present in the *A. tribuloides* root extract, including phenolic compounds, flavonoids, and saponins [22], could be responsible for the bioreduction of the Ag (I) ions and the capping of the resultant AgNPs.

### 3.2. Characterization of the Green Synthesized AgNPs

#### 3.2.1. UV–Visible Spectroscopy

The UV–visible spectroscopy, which enables us to measure the characteristic localized surface plasmon resonance (LSPR) absorption peak, was used to evidence the green synthesis of the AgNPs. The UV–vis spectra of the resulting reaction mixtures were determined at various time intervals (30, 60, 120, and 240 min) after starting the reaction (Figure 1). The spectra showed that the intensity of the LSPR absorption peak increased with the lapse of time, and its maximum was recorded at nearly 430 nm. This LSPR absorption peak was related to the formation and presence of the nearly spherical AgNPs [31]. 

#### 3.2.2. FTIR Spectroscopy

**Figure 2:**
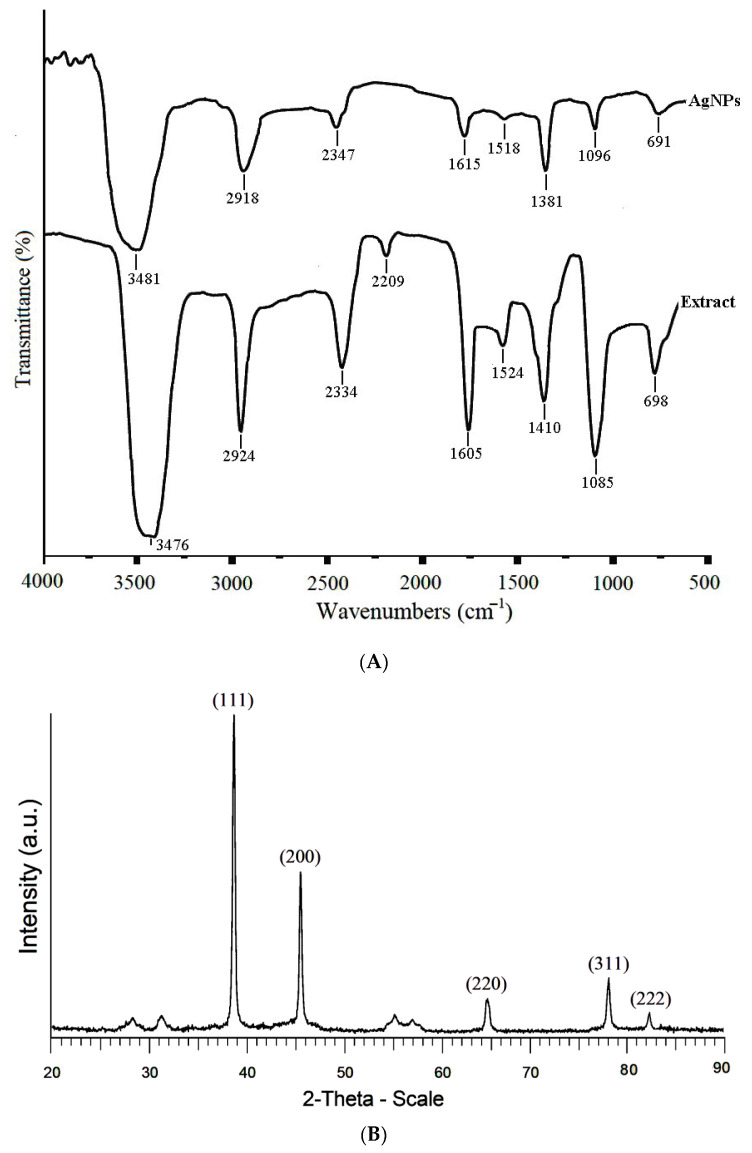
(**A**) The FTIR spectrum of the *A. tribuloides* root extract and the greenly synthesized AgNPs. (**B**) The XRD pattern of the AgNPs produced by using the *A. tribuloides* root extract.

The functional groups of the organic compounds of the *A. tribuloides* root extract that were responsible for reducing of the Ag (I) ions and capping the resultant AgNPs were identified by FTIR analysis (Figure 2A). The FTIR spectrum of the *A. tribuloides* root extract showed the absorption peaks at the following: 3476 cm^−1^, corresponding to the O-H stretching vibrations in alcohols and phenolic compounds [32]; 2924 cm^−1^, related to the C-H stretching vibrations of methyl groups [33]; 2334 cm^−1^, assigned to the H-C=O stretching vibrations in aldehydes; 2209 cm^−1^, corresponding to the C≡N stretching vibrations of nitriles [6]; 1605 cm^−1^, related to the C=O stretching vibrations in proteins; 1524 cm^−1^, assigned to the C-C stretching vibrations in the aromatic compounds [34]; 1410 cm^−1^, corresponding to the C-C stretching vibrations in alcohols, carboxylic acids, ethers, and esters [35]; 1085 cm^−1^, corresponding to C-O stretching vibrations in alcohols and phenols [36]; and 698 cm^−1^, corresponding to the C-H bonding vibrations of the aromatic compounds [37]. Figure 2 also shows the absorption peaks of the cleaned greenly synthesized AgNPs, which were at 3481, 2918, 2347, 1615, 1518, 1381, 1096, and 691 cm^−1^, and pointed out the stretching vibrations of O-H, C-H, H-C=O, C=O, C-C, C-C, C-O, and C-H bonds. These observations confirmed that the synthesized AgNPs were capped with the secondary metabolites (phenolic and flavonoid compounds) of the *A. tribuloides.* These components prevent the nanoparticles from the aggregation and stabilize their structure. By comparing the FTIR spectra of the *A. tribuloides* root extract and the greenly synthesized AgNPs, it could be observed that some peaks acquired for the *A. tribuloides* root extract were also observed in the FTIR spectrum of the greenly synthesized AgNPs, but their regions were changed. The absorption peaks noted in the FTIR spectrum of the AgNPs at 3476, 2334, 1605, and 1085 cm^−1^, in the *A. tribuloides* root extract spectrum, were shifted to higher-frequency positions, while those at 2924, 1524, 1410, and 698 cm^−1^ were shifted to lower-frequency positions. These changes showed that the functional groups associated with these bands were responsible for the bioreduction of the Ag(I) ions and the stabilization of the resultant AgNPs.

#### 3.2.3. X-Ray Diffraction (XRD)

The crystalline nature of the greenly synthesized AgNPs was approved by using the XRD pattern analysis, as shown in Figure 2B. The main diffraction peaks were observed at the 2θ values of 38.6°, 46.4°, 64.8°, 77.9°, and 82.3°, which corresponded to the (111), (200), (220), (311), and (222) Miller indices, respectively. These indices indicated the face-centered cubic (FCC) structure of Ag based on the database of Joint Committee on Powder Diffraction Standards (JCPDS), file No. 04-0783. The maximum intensity in the diffractogram was at the 2θ value of 38.6°, likely due to the organic components of the *A. tribuloides* root extract, which were responsible for the bioreduction of the Ag (I) ions and the stabilization of the greenly synthesized AgNPs [38,39]. In agreement with the FTIR analysis, the XRD analysis showed that the plant-derived organic compounds were attached to the surface of the AgNPs. These compounds stabilized the nanoparticles structure and probably added the new characteristics to these nanoparticles. Based on the Debye–Sherrer formula, the average crystalline size of the synthesized AgNPs was 34.2 nm.

#### 3.2.4. Transmission Electron Microscopy (TEM)

**Figure 3:**
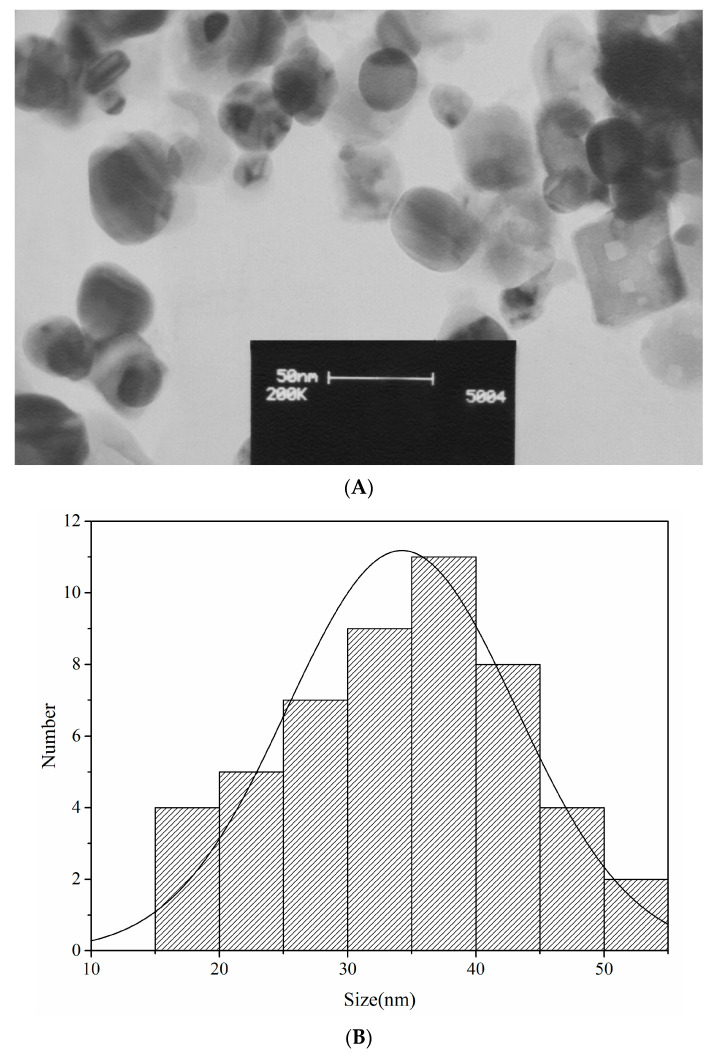
(**A**) The TEM image and (**B**) the particles size distribution of the AgNPs greenly synthesized by the *A. tribuloides* root extract acquired by using the TEM image (particles number = 50). Each bar shows the nanoparticles number with a certain size range.

The TEM analysis (Figure 3A,B) enabled us to establish that the morphology of the greenly synthesized AgNPs was spherical, with a broad distribution of size from 16.2 to 51.5 nm. In agreement with the size calculated by the XRD pattern, the average size of the AgNPs was 34.2 ± 8.0 nm.

### 3.3. In Vitro Biological Activities

#### 3.3.1. Total Phenolic and Flavonoid Contents 

**Figure 4:**
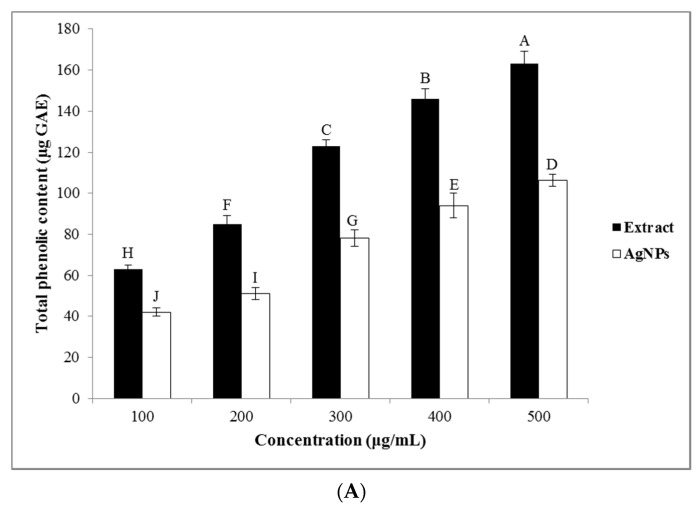

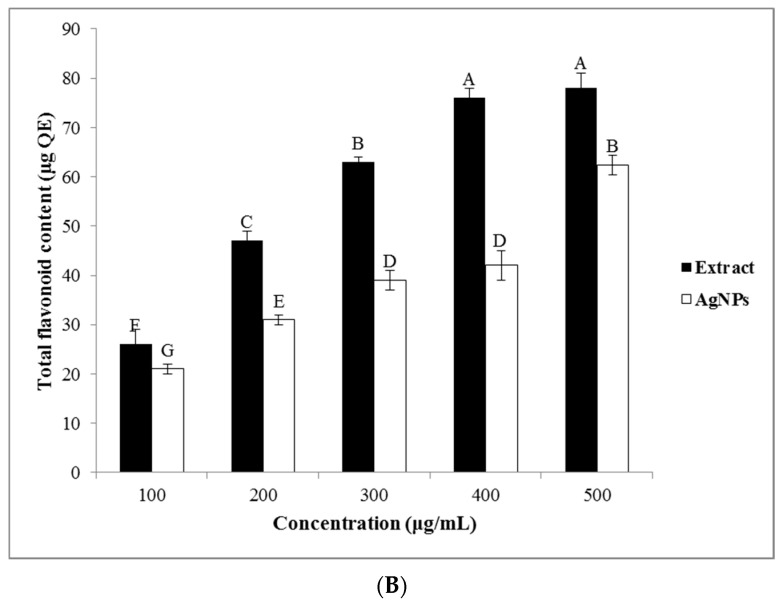
(**A**) Total phenolics and (**B**) total flavonoids content of the *A. tribuloides* root extract and the greenly synthesized AgNPs at various concentrations. Various letters represent significant differences (*p <* 0.05) between the *A. tribuloides* root extract and the greenly synthesized AgNPs at various concentrations, and also among various concentrations of each of the *A. tribuloides* root extract and the greenly synthesized AgNPs based on Duncan’s test.

The phenolic and flavonoid compounds are important secondary metabolites of plants for the bioreduction of the Ag (I) ions, and their amount in the plant extract can affect the reaction kinetic, shape, and size of the greenly synthesized NPs. The results showed that the TPC and the TFC of the *A. tribuloides* root extract and the greenly synthesized AgNPs were increased with their increasing concentration (Figure 4A,B). The analysis showed that the *A. tribuloides* root extract contained 163.2 ± 2 μg GAE of the TPC and 78.1 ± 3 μg QE of TFC at the highest concentration of the extract (500 μg/mL). The TPC and the TFC of the same concentration of the AgNPs were 106.4 ± 1 μg GAE and 62.4 ± 2 μg QE, respectively. The presence of these compounds on the synthesized AgNPs’ surface approved the role of the polyphenolic compounds as the reducing agents in the AgNPs production [40]. 

#### 3.3.2. Antioxidant Properties

**Figure 5:**
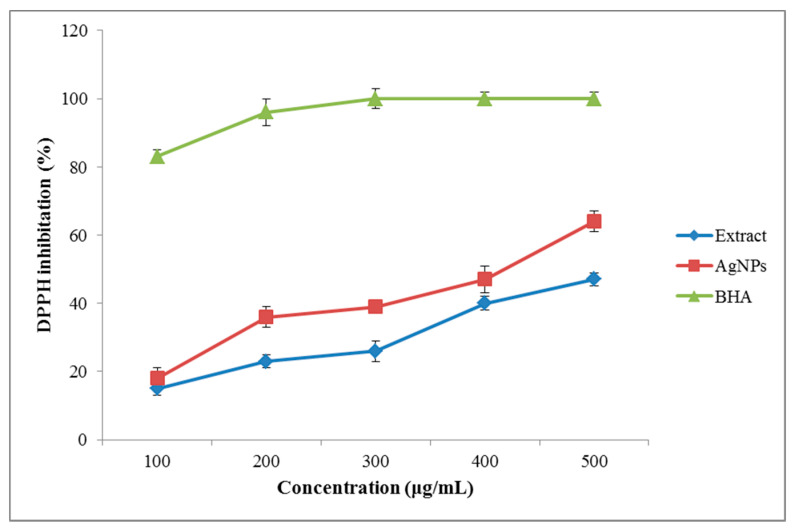
The DPPH scavenging activity of the *A. tribuloides* root extract and the greenly synthesized AgNPs at different concentrations.

The free-radical scavenging activity of the *A. tribuloides* root extract and the greenly synthesized AgNPs was evaluated by using the DPPH radical scavenging method (Figure 5). According to the results, the DPPH scavenging activity of the extracts and the fabricated AgNPs increased with their increasing concentration. The inhibition percentage for the highest concentration (500 μg/mL) of the *A. tribuloides* root extract was 47%, while this amount for the greenly synthesized AgNPs was 64%. The highest antioxidant properties of the AgNPs in comparison to the extract were likely associated with the adsorption of the bioactive compounds over the spherically shaped AgNPs [41]. It could also be attributed to the concurrent activity of the polyphenols as the antioxidant compounds and the AgNPs as a catalyst agent [42].

#### 3.3.3. Antibacterial Properties

##### Disc Diffusion Method 

The results of the antibacterial activity of the *A. tribuloides* root extract and the greenly synthesized AgNPs at similar concentrations (500 μg/mL) against the Gram-negative bacterial strains (*E. coli* and *Sh. flexneri*) and the Gram-positive bacterial strains (*B. cereus* and *S. aureus*) under the disc diffusion method are shown in Table 1. It was found that the *A. tribuloides* root extract and the greenly synthesized AgNPs had a considerable antibacterial effect toward the studied bacteria strains. The achieved AgNPs showed, however, the higher inhibition activity against the Gram-negative bacteria than the Gram-positive bacteria strains, while the *A. tribuloides* root extract had the higher inhibition activity on the Gram-positive bacteria in comparison to the Gram-negative bacteria strains. The AgNPs showed the higher inhibition activity against both the Gram-negative and Gram-positive bacteria than the *A. tribuloides* root extract. This different susceptibility of the bacterial strains could be associated with the differences in their cell-wall structure. The cell walls of the Gram-positive bacteria are composed of a thick peptidoglycan layer (approximately 20–80 nm), while this layer in the Gram-negative bacteria is thinner (approximately 7–8 nm), located between the two layers of the periplasmic space and covered with an outer membrane formed by the liposaccharides components having the negative charge. Thus, the AgNPs, having the positive relative charge, could be better attached to their cell-wall surface and penetrate through it [43]. In addition, the binding of the AgNPs to the proteins in the bacterial cell wall could cause its damage and be responsible for the leakage of the cellular contents, finally resulting in the bacterial death [44]. After the penetration of the AgNPs into the cell wall, they might also enter the cytoplasm of bacteria and interact with the intercellular biomolecules and structures, such as DNA, proteins, enzymes, and ribosomes. These processes can lead to the damage of the intracellular structures and to cell death [45].

##### Determination of the Minimum Inhibitory Concentration (MIC) and Minimum Bactericidal Concentration (MBC)

To evaluate the susceptibility of the bacterial strains to the *A. tribuloides* root extract and the greenly synthesized AgNPs, the MIC and MBC were determined (Table 1). The MIC values of these AgNPs and the *A. tribuloides* root extract differed remarkably. The greenly synthesized AgNPs exhibited the best antibacterial properties on all studied bacteria strains, with the MIC values ranging from 15.6 to 62.5 µg/mL. The corresponding values for the *A. tribuloides* root extract ranged between 125 and 250 µg/mL. The MBC values of the obtained AgNPs and the *A. tribuloides* root extract ranged from 31.2 to 125 µg/mL and from 250 to 500 µg/mL, respectively. The higher antimicrobial activity of the greenly synthesized AgNPs was possibly associated with their relatively large surface area, which makes them more available for the surface interactions and improves their bactericidal effects [46]. It could also be due to the spherical morphology and the small size of the AgNPs that allow them to easily cross the bacteria cell wall and destroy the cells [47].

##### Time-Kill Test of the Greenly Synthesized AgNPs

**Figure 6:**
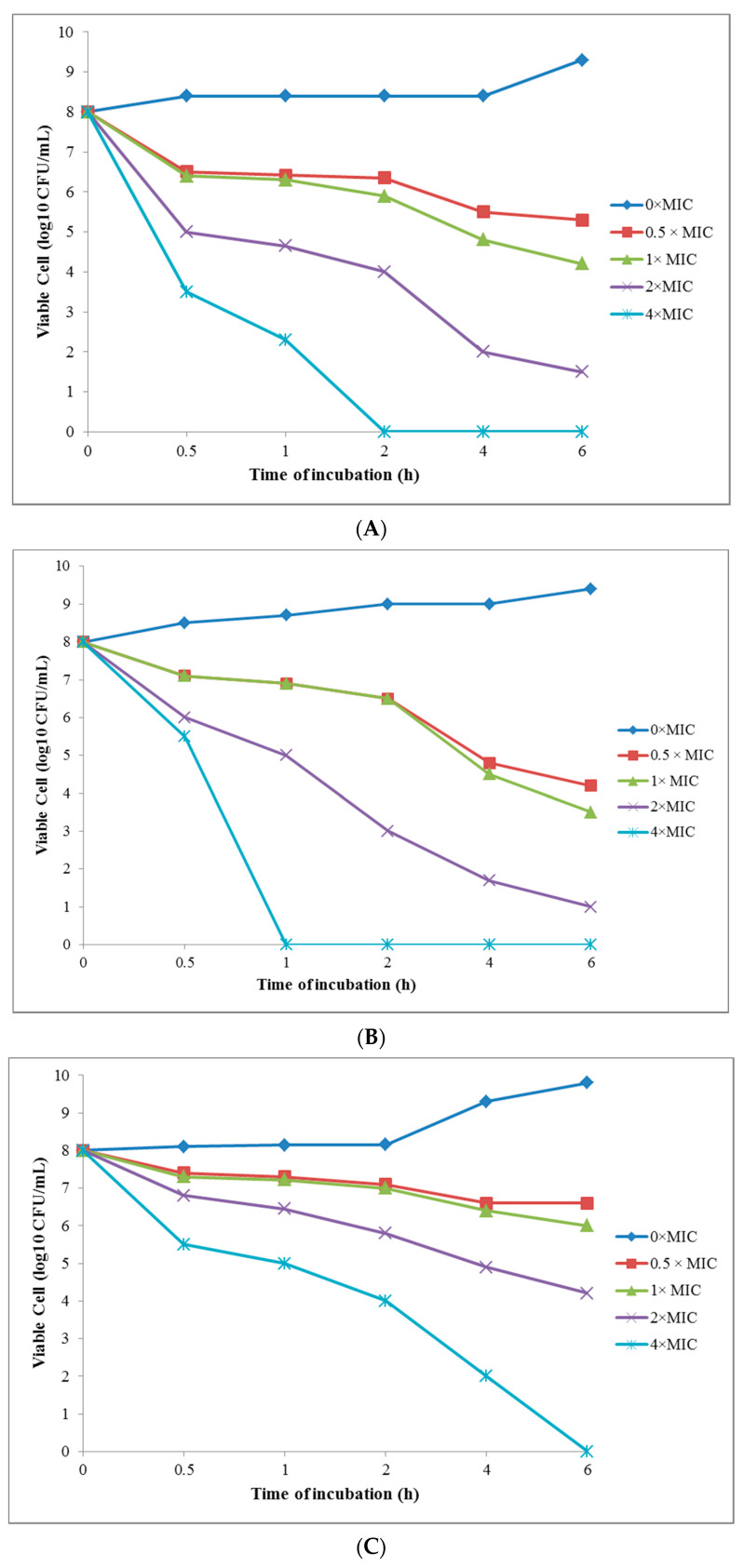

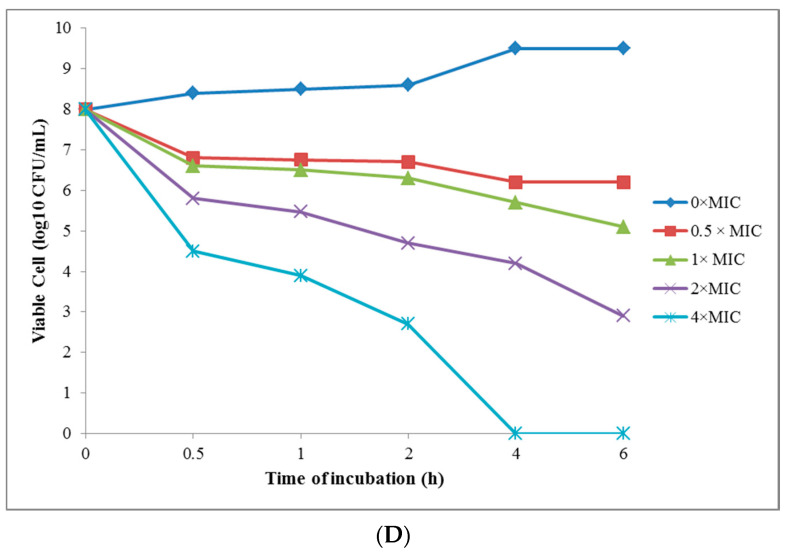
(**A**) The time-kill curve plots for *Escherichia coli* (**B**) *Shigella flexneri* (**C**) *Bacillus cereus*, and (**D**) *Staphylococcus aureus*, following the exposure to the greenly synthesized AgNPs at 0 × MIC, 0.5 × MIC, 1 × MIC, 2 × MIC, and 4 × MIC.

In the current study, the time-killing test was done to investigate the relation between the concentration of the greenly synthesized AgNPs with their killing properties on the studied bacteria strains. The time-kill curve test showed that the AgNPs killed *E. coli*, *Sh. flexneri*, *B. cereus*, and *S. aureus* at 4 × MIC, after 2, 1, 6, and 4 h, respectively (Figure 6A–D). On the other hand, the greenly synthesized AgNPs had the high antibacterial effects on the Gram-negative bacteria and, hence, could be a good antibacterial agent for this purpose. 

#### 3.3.4. Anti-Inflammatory Effects 

##### Inhibition of the Protein Denaturation

**Figure 7:**
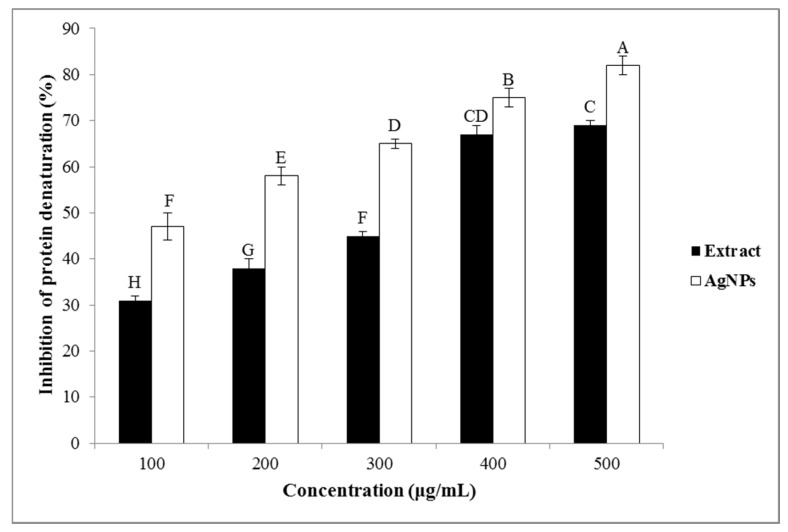
The anti-inflammatory effect of the *A. tribuloides* root extract and the greenly synthesized AgNPs. Various letters represent significant differences (*p <* 0.05) based on the Duncan’s test.

The protein denaturation is a perfectly documented reason for the inflammation in conditions as rheumatoid arthritis [48]. The prevention of the protein denaturation is the main mechanisms of action of non-steroidal anti-inflammatory drugs (NSAIDs) [49]. Therefore, the ability of the studied extract and the greenly synthesized AgNPs to prevent the denaturation of proteins could be responsible for their anti-inflammatory properties. As can be seen in Figure 7, both the *A. tribuloides* root extract and the greenly synthesized AgNPs produce a considerable anti-inflammatory activity in a dose-dependent manner. Its value for the highest concentration (500 μg/mL) of the *A. tribuloides* root extract was 69%, while this amount for the synthesized AgNPs was 82%. It was observed that the greenly synthesized AgNPs had the higher anti-inflammatory effects as compared to those for the *A. tribuloides* root extract. The results of the current study indicated that the obtained AgNPs were capped by the secondary metabolites of the *A. tribuloides* root extract. It was previously suggested that the secondary metabolites of the plant extracts can inhibit the lysosomal components release of the neutrophils at the inflammation location [50]. The lysosomal compounds are the proteinases and bactericidal enzymes that cause the further inflammation and the tissue damage after the extracellular release [51].

## 4. Conclusions

The current study presented the very simple, fast, affordable, and environmentally friendly green synthesis of AgNPs by the *A. tribuloides* Delile. root extract as a bioreducing and capping agent. The synthesized AgNPs showed an LSPR absorption band at 430 nm. The crystal structure of the greenly synthesized AgNPs was approved by using the XRD analysis. The FTIR analysis pointed out the presence of the plant metabolites on the surface of the AgNPs. The TEM analysis indicated that the synthesized AgNPs were spherical, with a distribution of size from 16.2 to 51.5 nm. The AgNPs fabricated by using the *A. tribuloides* root extract according to the procedure proposed in the present work have potent antioxidant, antibacterial, and anti-inflammatory properties, which were remarkably better than those established for the extract itself. Therefore, the greenly synthesized AgNPs can be used for biomedical applications, as a new drug combination.

## Figures and Tables

**Table 1:** The inhibition zone, minimum inhibitory concentration (MIC), and minimum bactericidal concentration (MBC) of the *A. tribuloides* root extract and the greenly synthesized AgNPs against the tested bacterial strains.

Bacteria	Diameter of Inhibition Zone (mm)			MIC (μg/mL)		MBC (μg/mL)	
	Gentamicin (10 µg/Disk)	AgNPs	Extract	AgNPs	Extract	AgNPs	Extract
*Escherichia coli*	10	24	8	31.25	250	62.5	500
*Shigella flexneri*	12	27	10	15.62	250	31.25	500
*Bacillus cereus*	15	16	13	62.5	125	125	250
*Staphylococcus aureus*	18	19	15	62.5	125	125	250
